# Experimental Releases of Pupal Parasitoids to Control *Piophila casei* (L.) (Diptera: Piophilidae) in Ham Production

**DOI:** 10.3390/insects17090908

**Published:** 2026-08-30

**Authors:** Diletta Missere, Riccardo Girodo, Antonio Martini, Niccolò Patelli, Giovanni Burgio

**Affiliations:** 1 Department of Agricultural and Food Sciences, University of Bologna, 40127 Bologna, Italy; riccardo.girodo2@unibo.it (R.G.); antonio.martini@unibo.it (A.M.); giovanni.burgio@unibo.it (G.B.); 2 Bioecology S.r.l., Via Della Corte 4, Corte Tegge, 42025 Reggio Emilia, Italy; patelliniccolo88@gmail.com

**Keywords:** biological control, pupal parasitoids, parasitism rate, stored product pests, Hymenoptera, Pteromalidae, Diptera Piophilidae

## Abstract

The use of biological control within an integrated pest management approach in stored food production is expected to increase, due to both the limitations of chemical methods and the rising focus on environmental sustainability. However, comprehensive biological control strategies for animal-derived products like meats, fish, and cheeses are still lacking. Dry-cured ham is an example of a preserved animal product that can be infested by various arthropods, such as *Piophila casei* (L.) (Diptera: Piophilidae), when stored. This study aimed to assess the effectiveness of releasing the pupal parasitoids *Pachycrepoideus vindemiae* (Rondani) (Hymenoptera: Pteromalidae) and *Muscidifurax raptor* Girault and Sanders (Hymenoptera: Pteromalidae) in controlling this dangerous fly in a ham production facility in Northern Italy. Data on total parasitism, emergence rates, sex ratio, and longevity were collected for *M. raptor* and *P. vindemiae* during the biological control experiments. It was found that both parasitoids could successfully parasitize pupae of *P. casei* in ham production, avoiding ham contamination. While the longevity and the sex ratio data did not show a significant difference between the two parasitoids, *P. vindemiae* demonstrated a significantly higher total parasitism and emergence rate, suggesting it as a more promising candidate under the conditions of this study. An assessment of the potential use of these parasitoids in a ham production facility is discussed.

## Simple Summary

The protection of stored foods from insect pests is an increasing challenge, especially as the use of chemical insecticides becomes more restricted for environmental and health reasons. Dry-cured ham, a traditional and high-value product, can be attacked by *Piophila casei* (L.) whose larvae feed on the meat, causing serious economic losses and food safety concerns. This study investigated whether two pupal parasitoids could be used as a natural and safe method to control this pest in a ham production facility in Northern Italy. The results showed that both species were able to successfully attack the pest without contaminating the ham. However, one of the two species showed higher total parasitism and emergence rates. These findings suggest that biological control using parasitoids could be a promising alternative to chemical treatments in ham production and deserve further investigation under commercial conditions, helping to protect food quality while supporting more sustainable and environmentally friendly practices.

## 1. Introduction

Historically, biological control in agriculture was often regarded as a “last resort”, typically adopted only after conventional insecticides had failed [1,2]. However, increasing concerns about the environmental and human health impacts of chemical insecticides, together with the development of insecticide resistance and stricter pesticide regulations, have progressively shifted this perception. Today, biological control is widely recognized as a key component of integrated pest management and, in some cropping systems, such as greenhouses, it has become the preferred pest control strategy [3]. Similarly, the use of biological control in stored-product protection has gained increasing attention because of the limitations associated with chemical control methods [4]. Nevertheless, despite scientific advances, its practical adoption in stored-product systems remains limited to relatively few sectors [5,6]. Most biocontrol strategies target moths in bakeries, food-processing industries, and retail trade, and beetles in stored grains. The broader category of animal-derived products, including meats, fish, and cheeses, still lacks comprehensive biological control strategies and research and commercial development in this area remain limited compared with grain storage systems. This gap can be attributed, at least in part, to the very low tolerance for insect presence in foods intended for human consumption and to concerns regarding the deliberate release of natural enemies in food-processing facilities [7].

Among the preserved animal-derived products, dry-cured ham stands out for its significant economic value, particularly in Italy, where renowned PDO (Protected Designation of Origin) products such as Prosciutto di Parma contribute substantially to the agri-food sector [8]. Dry-cured ham is made by salting the hind leg of a pig and aging it under specific temperature and humidity conditions for periods ranging from 2 months to 2 years [9]. The storage of dry-cured ham has been shown to favor infestation by *Piophila casei* Linnaeus (Diptera: Piophilidae) [10,11].

*Piophila casei* is characterized by a high reproductive rate. At 25 °C, a temperature comparable to that encountered during the final stages of dry-cured ham production [9], females can produce up to 250 eggs during their lifetime, allowing populations to increase rapidly [12,13]. Consequently, infestations are most frequently observed during the later stages of the curing process, when environmental conditions favor the development of the fly. After hatching, larvae penetrate deeply into the substrate and feed intensively, producing internal galleries that are not immediately detectable from the external surface, along with putrid odors [11,14]. As larval development progresses, this damage becomes increasingly evident, rendering the product unsuitable for sale and resulting in substantial economic losses [12]. At the end of larval development, the larvae leave the feeding galleries by dropping from the substrate or by performing a characteristic skipping behavior, in which the body is curled into a C-shape and suddenly released to propel the larva into the air. This behavior is the origin of the common name “cheese skipper” [11].

Infestations by *P. casei* pose several challenges for the development and implementation of prophylactic and control strategies. Sanitation is the primary measure against these infestations, but it is not always sufficient on its own, since larvae leave the food substrate to pupate in the flooring, where crevices provide refuges that routine cleaning does not readily reach. Chemical control methods, including the use of fumigants such as phosphine and, historically, methyl bromide, as well as contact insecticides (e.g., pyrethroids and organophosphates), have been commonly employed in ham production facilities [10,15]. However, the effectiveness of these compounds has progressively declined owing to the development of insecticide resistance in *P. casei*, as well as increasing regulatory restrictions and concerns about environmental impact, food safety, and operator health [16,17,18]. Non-chemical techniques such as extreme-temperature treatments [19], X-ray irradiation [20], and essential oil treatments have been explored [14], but their effectiveness and potential impact on the organoleptic characteristics of the ham are unclear. Finding a natural method to reduce infestation without altering the ham sensory profile is a pressing issue [21,22].

Agricultural successes with biological control have inspired us to explore the potential of using beneficial arthropods for controlling *P. casei* infestation in dry-cured ham production. The characteristic behavior by which *P. casei* larvae leave the food substrate and pupate in the flooring has motivated us to study pupal parasitoids as a means of control, which could prevent product contamination and the associated costly recalls [11]. Building on this rationale, we selected two parasitoid species with documented activity against dipteran pupae to test their efficacy against *P. casei* under field conditions.

The research was conducted in a ham factory in Northern Italy, where biological control experiments were carried out in customized cages using *Pachycrepoideus vindemiae* (Rondani) (Hymenoptera: Pteromalidae) and *Muscidifurax raptor* Girault and Sanders (Hymenoptera: Pteromalidae). Both species are ectoparasitic parasitoids [23,24] that parasitize the pupae of a wide range of dipteran hosts [25].

*Pachycrepoideus vindemiae* has been investigated as a biological control agent of *Drosophila suzukii* Matsumura (Diptera: Drosophilidae) [26], whereas *Muscidifurax raptor* has been widely used against synanthropic flies, particularly *Musca domestica* Linnaeus (Diptera: Muscidae) [27]. However, concerns regarding their broad host range have limited their use, as they may attack non-target species, including beneficial insects such as pollinators [28]. Such concerns are not applicable to the present case, as the parasitoids were confined within enclosed experimental units inside a dry-cured ham production facility, where non-target insect communities are minimal. Nevertheless, the selection of these parasitoid species was based on previous reports of their ability to parasitize *P. casei* pupae [29], together with our own preliminary laboratory evaluations, which confirmed this ability under controlled conditions and indicated their potential suitability as biological control agents [11].

Both these reports, however, refer to laboratory conditions. No previous study has released these parasitoids inside a working dry-cured ham production facility, and it therefore remains unknown whether the parasitization recorded in laboratory arenas translates into measurable parasitism under production conditions. Curing rooms differ from laboratory arenas in ways that are likely to affect parasitoid performance: they are large, structurally complex, and largely dark; the host pupae are dispersed over the flooring rather than confined to arenas; and routine production activities are carried out during the trial.

Parasitoids were released using an inundative approach, and data on total parasitism (TP), emergence rates (ER), emergence success (ES), fly emergence rate (FER), pupal mortality rate (PMR), sex ratio (SR) and longevity (LO) of the parasitoids were collected to evaluate the potential of these natural enemies to control *P. casei.* The study was designed as a preliminary evaluation of feasibility under commercial curing room conditions, intended to indicate whether larger-scale, replicated trials are justified.

## 2. Materials and Methods

### 2.1. Experimental Cages and Study Site

**Figure 1:**
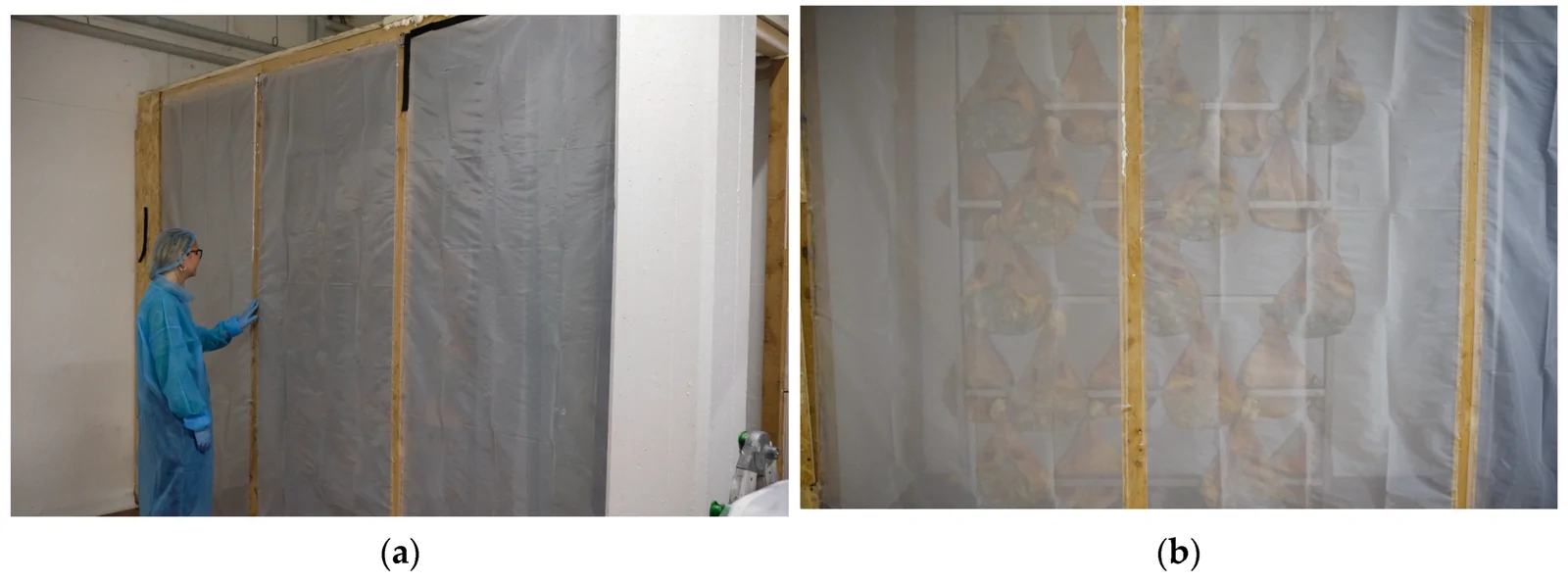
Experimental cages used in the study. (**a**) External view of one of the experimental cages. (**b**) Internal view showing a portion of the dry-cured hams hanging inside the cage.

We conducted a biological control experiment in three customized cages built in an underground curing room at the ham factory to simulate biological control under commercial ham production conditions. The curing room remained dark under normal operating conditions and was illuminated only during routine activities performed by the facility staff; however, the timing and duration of artificial lighting were not monitored. Each customized cage was selected for releasing one species of parasitoids (*M. raptor* or *P. vindemiae*), with the exception of one control cage, which received no parasitoid releases and was included exclusively to confirm the absence of pre-existing parasitoids within the experimental facility before the trials commenced. The control was not designed for comparative analysis of host population dynamics across treatments. Analysis of host population dynamics was omitted, as independent activities within the facility during the experimental period could have influenced host abundance, thus precluding a reliable evaluation of population trends. Each cage was 2.5 m^2^ (2.5 m height) and constructed of wooden frames fitted with 160 μm mesh walls to prevent the insects from escaping. The edges and the base were sealed with polyurethane foam to ensure insect containment. Twenty-five hams (approximately 9 kg each) were placed inside each cage, hung, and processed in the same way as hams intended for sale in the curing room (Figure 1).

### 2.2. Maintenance and Preparation of Insects Used in the Biological Control Experiment

The colonies of *P. casei*, *P. vindemiae*, and *M. raptor* used for the biological control experiment were maintained in a walk-in climatic chamber in the laboratory of the Department of Agricultural and Food Sciences, University of Bologna, under standardized laboratory conditions (25 ± 1 °C, 50–60% RH and a 16 L:8 D photoperiod). The rearing temperature was selected based on the temperatures recorded in the commercial ham curing room during the three parasitoid release periods (22.7–23.7 °C), while also ensuring standardized conditions for colony maintenance. The humidity range corresponds to the control range of the walk-in climatic chamber rather than to a deliberate variation, and falls within the values typical of curing rooms during the ageing of dry-cured ham [9]. Adult insects were kept in Plexiglas^®^ (Leroy Merlin, Bologna, Italy) cages provided on each side with an opening covered with plastic mesh for ventilation. The pest colony was supplemented monthly with field-collected specimens. To limit inbreeding, the *P. vindemiae* colony was supplemented each summer with wild individuals obtained by exposing Petri dishes containing *P. casei* pupae as sentinel hosts in the garden of the Department for three days; emerging parasitoids were identified before being introduced into the colony. Individuals of *M. raptor* were not collected: new individuals were obtained periodically from Bioecology S.r.l. (Reggio Emilia, Italy), the commercial supplier of the original colony.

#### 2.2.1. Piophila Casei

The flies were fed with fresh ham to keep the diet consistent with that used in the biological control experiment. To obtain simultaneous emergence of adult flies, 1-day-old pupae were removed from the colony and confined in another bug dorm. The adults obtained were maintained only with a solution of 20% sugar dissolved in water to prevent oviposition until they were used in the experiment.

#### 2.2.2. Muscidifurax Raptor and *Pachycrepoideus vindemiae*

The *P. vindemiae* colony has been maintained at the Department since 2020, where it was founded from field-collected individuals and identified following Bouček and Rasplus [30]. The morphological characters used for identification are illustrated in Missere et al. [11]. The *M. raptor* colony was started from adults emerging from parasitized *M. domestica* pupae supplied by Bioecology S.r.l., which rears this species commercially.

For parasitoid rearing, the wasps were fed daily with honey drops applied to small pieces of paper and a dispenser with a solution of 20% sugar dissolved in water. To obtain synchronized adult wasps to release in the experiment, the parasitoids were provided with *P. casei* pupae that were at least 3 days old. Host diet has been considered as a potential factor affecting the parasitism rate in the field [31]. Therefore, only host pupae obtained from fresh ham (Prosciutto di Parma PDO, aged 18 months) were used. The parasitoids had been reared on ham-fed hosts for five generations before the first release. Each pupa was confined to a single glass vial (3.5 × 12.5 cm). These conditions allowed a new parasitoid generation to be produced in 20–25 days. Newly emerged adult parasitoids were collected daily, counted, and sexed.

### 2.3. Artificial Infestation of Customized Cages

The customized cages were artificially infested with *P. casei* in order to compare the performance of the two parasitoids at the same initial pest density and standard conditions in the biological control trials. Each customized cage was manually infested with 100 female and 100 male *P. casei* adults obtained from the laboratory culture to ensure sufficient host availability for parasitoid activity. To minimize the risk of failed pest establishment, two releases were made at each cage. The second release consisted of 75 females and 75 males, for a total of 175 females and 175 males per cage.

### 2.4. Parasitoid Releases

In each customized cage, a single parasitoid species was released: *M. raptor* was released in cage 1, and *P. vindemiae* in cage 2. Cage 3, where parasitoids were not released, was considered the control treatment. Releases were conducted when the first fly pupae appeared on the bottom of the customized cages (i.e., approximately 14 days following the ham infestation).

The parasitoids were transported from the laboratory to the ham factory in vials with perforated caps, containing wet cotton for humidity and honey to feed the parasitoids. For each species (*M. raptor* and *P. vindemiae*), 200 individuals (100 females and 100 males; female:male ratio 1:1) were released per release event. Parasitoids were released together within 72 h of emergence, while still unmated, allowing us to assess their ability to locate mates within the larger, more spatially complex environment of the customized cage. This is an important measure, since mating success ultimately determines the number of offspring produced and, in turn, the parasitism potential of that offspring. Successful mating was subsequently assessed indirectly through the progeny sex ratio (SR), because in both arrhenotokous parasitoid species unmated females produce only male offspring [32].

The vials with parasitoids were placed on the floor of the cages. Once the lids were removed, the parasitoids dispersed into the environment. The timing for releasing the parasitoids was based on the development time of laboratory-reared parasitoids, and *M. raptor* and *P. vindemiae* were released roughly every 21 days.

Three releases were conducted at each cage to ensure a sufficient parasitoid population throughout the experiment, taking into account the emergence performance of both species on *P. casei* observed under laboratory conditions [11].

### 2.5. Estimation of Piophila casei Parasitization by the Two Parasitoids

To assess the effectiveness of parasitoids in customized cages, we monitored parasitism level by collecting, storing, examining, and eventually dissecting *P. casei* pupae.

#### 2.5.1. *Piophila casei* Pupae Sampling

*Piophila casei* pupae were collected three times after each release (7, 14, and 21 days post-release). At each sampling event, all pupae present in each cage were counted. To ensure host availability for parasitoids between subsequent sampling events, 20 pupae were intentionally left in each cage; the remaining pupae constituted the population size (N) used for sample size estimation (n). To collect a representative sample of pupae, we used the formula proposed by Daniel (1995) [33].

n = [z^2^ p(1 − p)/e^2^]/1 + [z^2^ p(1 − p)/e^2^N], (1)

N is the population size, z is the z-score, e is the margin of error, and p is the expected proportion. A confidence level of 95%, a margin of error of 5%, and an expected proportion of 50% were assumed for sample size estimation. Consequently, the number of pupae collected varied among sampling dates (Table 1). Because this formula includes a finite population correction and takes N as an input, the sample size n it returns is a function of N. Variation in the number of pupae collected across sampling dates is therefore a direct consequence of applying the formula. The 20 pupae left in each cage were excluded from N before the formula was applied and did not enter the calculation.

#### 2.5.2. Storage of Pupae and Estimation of Parasitoids Performance

The *P. casei* pupae collected from each customized cage were stored separately in Eppendorf tubes kept in the laboratory at 25 ± 1 °C, 50–60% RH and 16 L:8 D photoperiod. Pupae collected at each weekly sampling were processed separately, allowing the estimation of parasitization parameters over time. The following parameters were recorded: total parasitism (TP), progeny emergence rate (ER), emergence success (ES), fly emergence rate (FER), pupal mortality rate (PMR), progeny sex ratio (SR), and progeny longevity (LO) [11].

#### 2.5.3. Dissection of Pupae

To obtain precise data on parasitization, pupae from which no parasitoid or *P. casei* adult had emerged after at least one month were transferred to Eppendorf^®^ (Eppendorf SE, Amburgo, Germania) tubes containing a 70% ethanol solution and subsequently dissected. This observation period exceeds the 20 to 25 days required for a new parasitoid generation to be completed under the same conditions, so that failure to emerge could not be attributed to incomplete development.

Pupae were fixed to Petri dishes using a 1.5-mm-thick double-sided foam mounting tape (Pattex Millechiodi Tape^®,^ Henkel Italia S.p.A., Milano, Italia), which ensured that the pupae remained firmly in place during dissections. We carefully opened the pupal operculum with microdissection forceps. Then, we cut one side of the pupal case to remove it completely and lifted the midsection to expose its contents. Subsequently, we positioned a soft bristle brush under the exposed contents and examined them under a stereomicroscope. The examination was done to detect for pre-imaginal stages of the parasitoids, the exuviae, and the adults.

### 2.6. Data Analysis

In this study, several indices that summarize the parasitization of *P. casei* by *M. raptor* and *P. vindemiae* were estimated. For the purpose of data analysis, the experimental unit was the cage, so that the number of true replicates is one per parasitoid species. The sampling units were the individual pupae collected on each sampling date. Individual pupae and sampling dates are subsamples of the same experimental unit and do not constitute independent replicates.

We calculated the emergence rate (ER) as the ratio of the number of emerging parasitoids to the total number of pupae. In order to obtain a more precise estimate of parasitization, the total parasitism (TP) was also calculated. This was estimated as the sum of the number of emerged parasitoids and the number of pupae found through dissection to contain a parasitoid (as egg, pupa, larva, or adult), divided by the total number of pupae.

Emergence success (ES) was calculated as the ratio of the total number of emerged adults (parasitoids and flies) to the total number of pupae. Fly emergence rate (FER) was calculated as the ratio of the number of emerged flies to the total number of pupae.

Pupal mortality rate (PMR) was calculated as the number of pupae from which neither parasitoids nor flies emerged, and that, upon dissection, were found to contain only a dead fly and no parasitoid, divided by the total number of pupae.

For each parasitoid release, data collected during the three sampling events were pooled by summing the numbers of pupae and their respective outcomes (emerged parasitoids, emerged flies, pupae found parasitized upon dissection but from which no parasitoid had emerged, and dead pupae). ER, TP, ES, FER, and PMR were then calculated as proportions based on these pooled counts, and binomial standard errors (BSE) were estimated for each parameter.

Pooling was adopted because the successive sampling dates within one cage are repeated observations on the same experimental unit rather than independent replicates, so that the pooled value summarises the data at the only level at which the observations are structurally independent. Each pooled index is the total number of pupae in the outcome category divided by the total number of pupae examined, so that each sampling date is weighted by its own sample size. The binomial standard error was calculated as SE = √[p(1 − p)/n], where p is the pooled proportion for the index considered and n is the total number of pupae on which that proportion was calculated. Because n increases with pooling, the standard errors derived from the pooled data are smaller than those that would be obtained from any individual sampling date.

To provide an overall estimate of parasitoid performance, the data from the three releases were further pooled for each parasitoid species, and the same indices were recalculated using the total numbers of pupae and corresponding outcomes recorded throughout the experiment.

Pearson’s chi-square (χ^2^) test was applied to the raw counts of pupae, arranged in 2 × 2 contingency tables in which each cell contained the number of pupae assigned to mutually exclusive outcome categories and the row totals were the numbers of pupae examined for each parasitoid species. The tests were performed for each of the parameters ER, TP, ES, FER and PMR, both across the entire dataset and within each release. The indices themselves are reported as descriptive summary statistics computed from the same counts and were not used as input to the tests. Statistical significance was set at *p* < 0.05.

To determine parasitoid longevity (LO), we measured the number of days from emergence to death. After emergence, adults were maintained under the laboratory conditions described in Section 2.5.2, without food or water, to simulate conditions typically encountered in ham production facilities, where adult parasitoids have little or no access to suitable food resources. Differences in longevity between the two parasitoid species were assessed using the Mann–Whitney U test. Statistical significance was set at *p* < 0.05.

Sex ratio (SR) was calculated as the proportion of females among the total offspring (females/[females + males]). Binomial standard errors were calculated for each estimate. A Pearson chi-square statistic was used to test the SR for the two wasp species. Statistical significance was set at *p* < 0.05.

Statistical analyses were performed using IBM SPSS Statistics (ver. 29).

## 3. Results

### 3.1. Estimation of Piophila casei Parasitization by the Two Parasitoids

In the control cage, which received no parasitoid releases, none of the pupae examined were parasitized, confirming the absence of pre-existing parasitoids in the experimental facility.

#### 3.1.1. Emergence Rate (ER)

**Figure 2:**
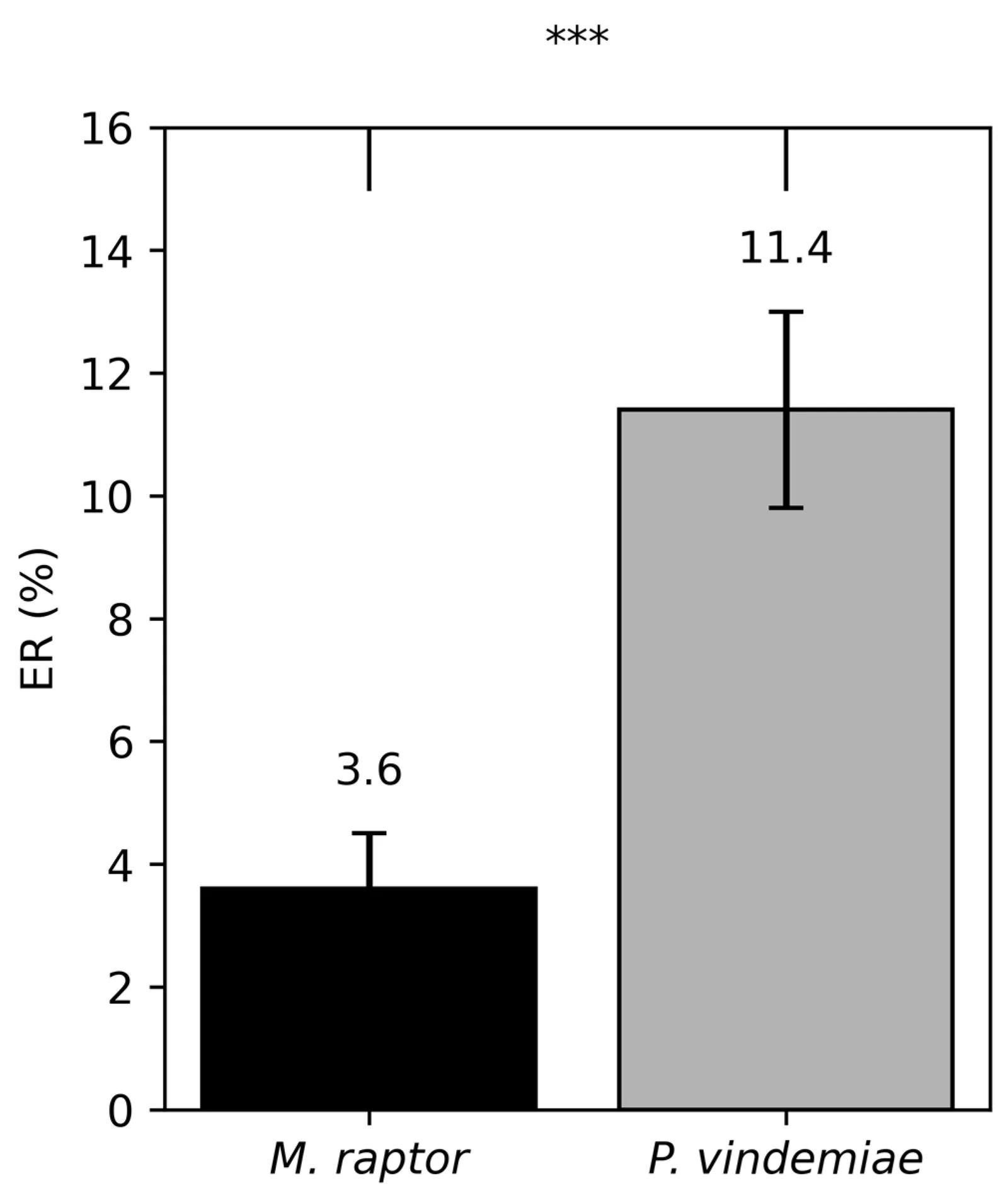
Comparison of the overall mean emergence rate (ER%) of *M. raptor* and *P. vindemiae*. Error bars represent the binomial standard error (BSE). Asterisks indicate significant differences between parasitoid species according to Pearson’s χ^2^ test (***, *p* < 0.001).

When data from the three releases were pooled, the overall ER was significantly higher in *P. vindemiae* (11.4 ± 1.6%; *n* = 413 pupae) than in *M. raptor* (3.6 ± 0.9%; *n* = 419 pupae) (χ^2^ = 18.35, df = 1, *p* < 0.001; Figure 2).

**Figure 3:**
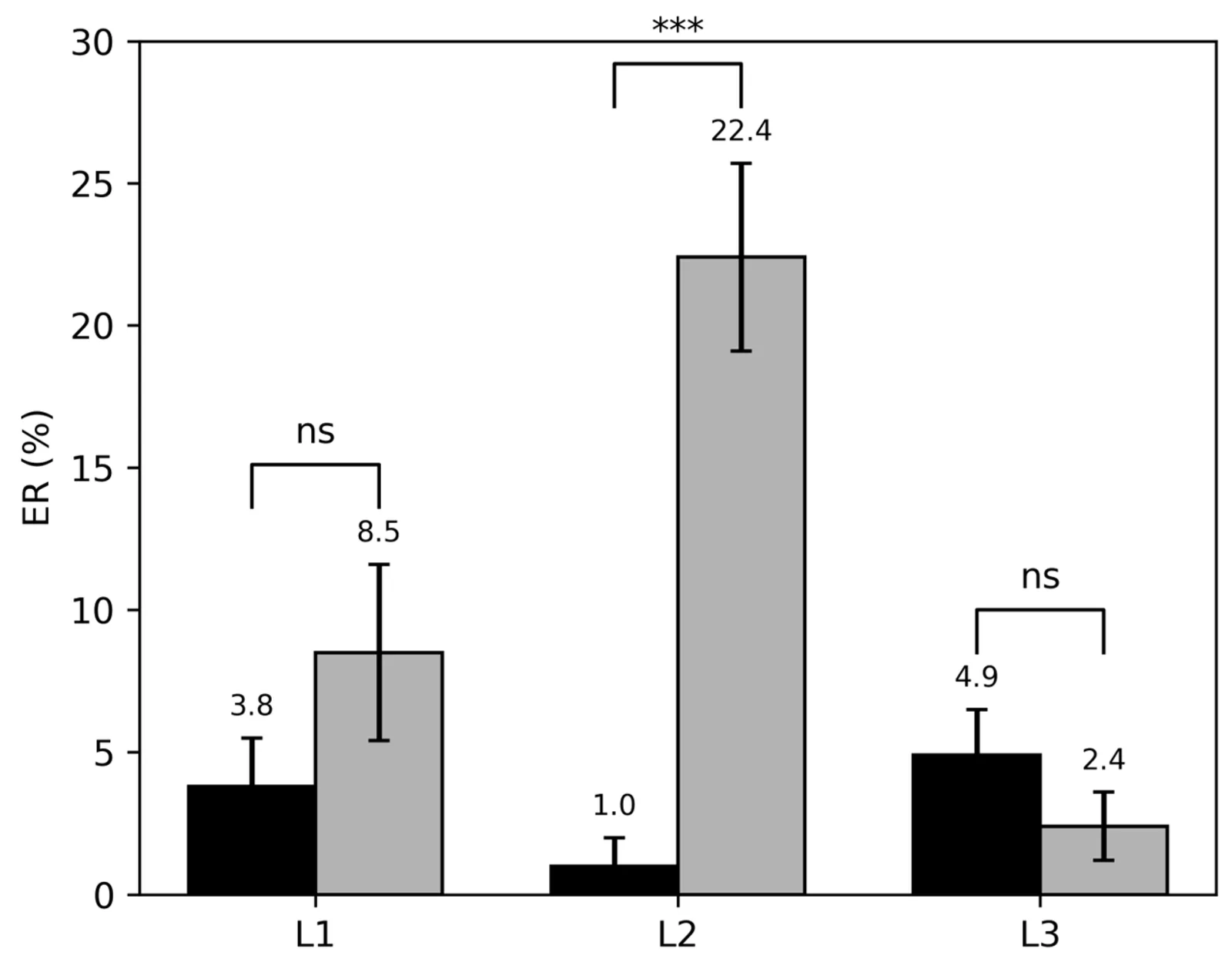
Comparison of the mean emergence rate (ER%) of *M. raptor* and *P. vindemiae* for each parasitoid release (L1–L3). Error bars represent the binomial standard error (BSE). Asterisks indicate significant differences between parasitoid species within each release according to Pearson’s χ^2^ test (***, *p* < 0.001; ns = not significant).

At the individual release level, the ER of *M. raptor* was 3.8 ± 1.7% after the first release (*n* = 130 pupae), 1.0 ± 1.0% after the second release (*n* = 104 pupae), and 4.9 ± 1.6% after the third release (*n* = 185 pupae) (Figure 3). In contrast, *P. vindemiae* showed ER values of 8.5 ± 3.1% after the first release (*n* = 82 pupae), 22.4 ± 3.3% after the second release (*n* = 161 pupae), and 2.4 ± 1.2% after the third release (*n* = 170 pupae).

A significant difference in ER between the two parasitoid species was detected only after the second release (χ^2^ = 24.08, df = 1, *p* < 0.001), whereas no significant differences were observed after the first release (χ^2^ = 2.07, df = 1, *p* = 0.150) or the third release (χ^2^ = 1.58, df = 1, *p* = 0.208).

#### 3.1.2. Total Parasitism (TP)

**Figure 4:**
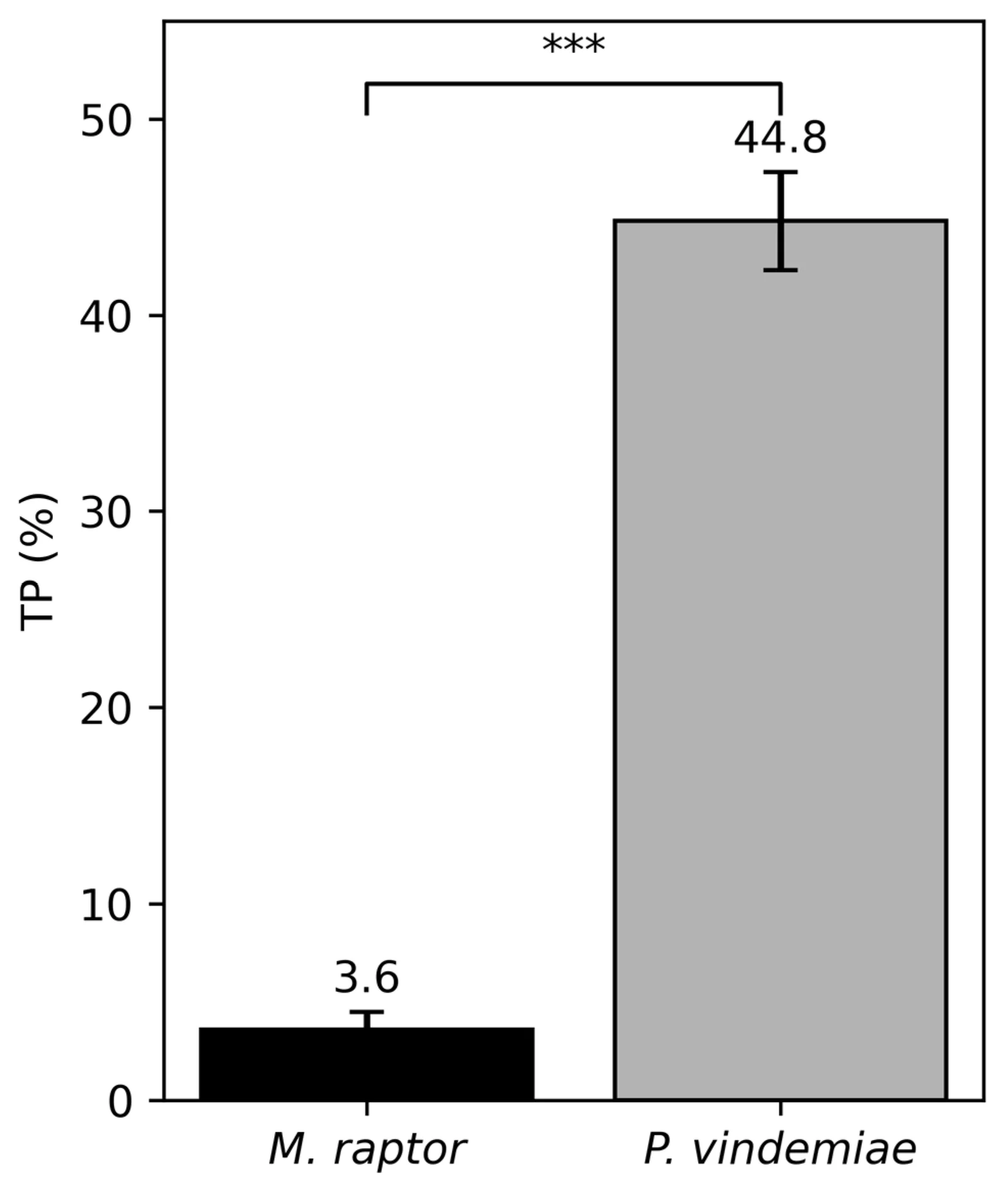
Comparison of the overall mean total parasitism (TP%) of *M. raptor* and *P. vindemiae*. Error bars represent the binomial standard error (BSE). Asterisks indicate significant differences between parasitoid species according to Pearson’s χ^2^ test (***, *p* < 0.001).

For *M. raptor*, TP corresponded to the ER, as dissection of host pupae did not reveal any parasitoids that had failed to emerge. When data from the three releases were pooled, the overall TP was 3.6 ± 0.9% (*n* = 419 pupae), which was significantly lower than that of *P. vindemiae* (44.8 ± 2.5%; *n* = 413 pupae) (χ^2^ = 193.48, df = 1, *p* < 0.001; Figure 4).

**Figure 5:**
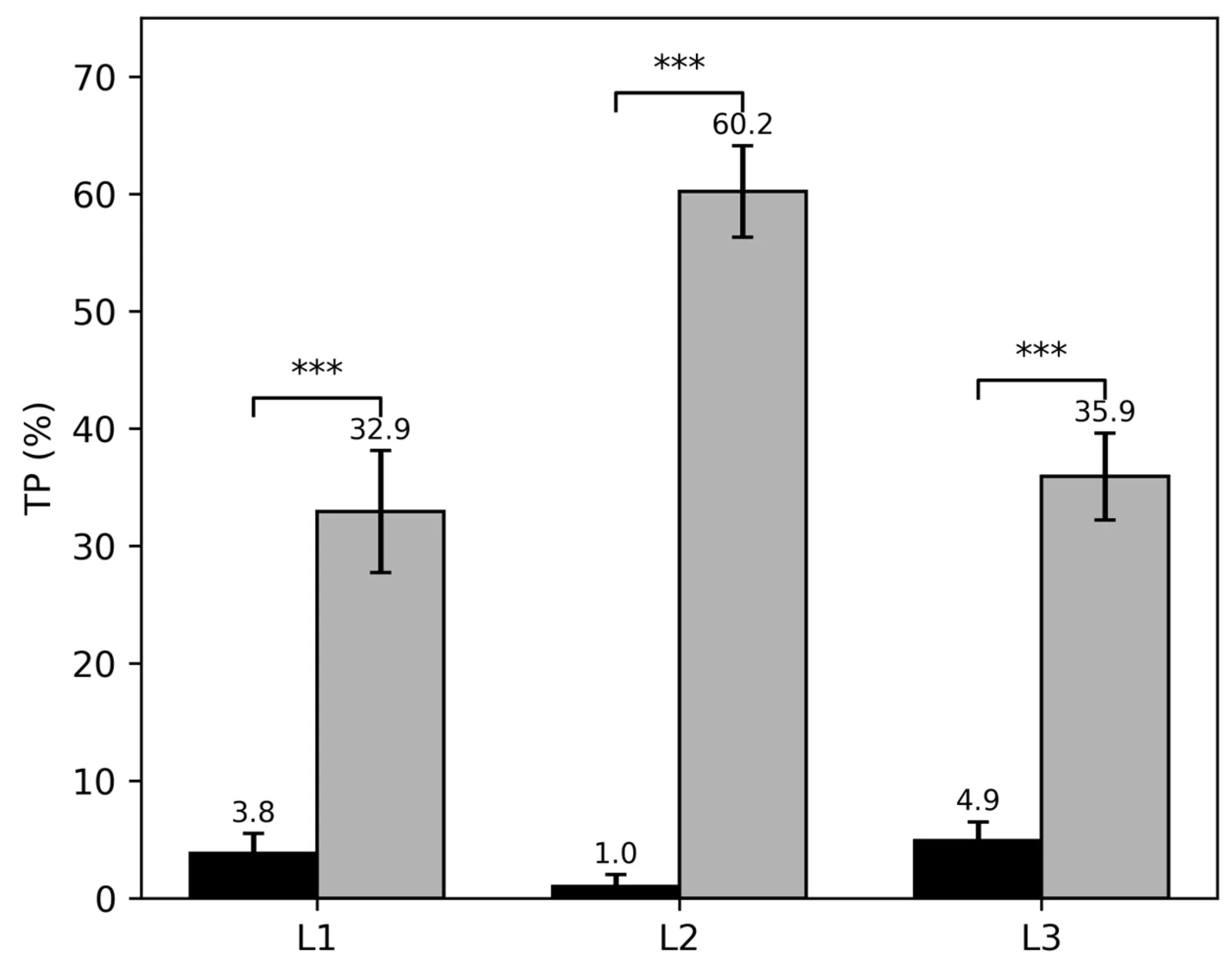
Comparison of the mean total parasitism (TP%) of *M. raptor* and *P. vindemiae* for each parasitoid release (L1–L3). Error bars represent the binomial standard error (BSE). Asterisks indicate significant differences between parasitoid species within each release according to Pearson’s χ^2^ test (***, *p* < 0.001).

At the individual release level, the TP of *M. raptor* was 3.8 ± 1.7% after the first release (*n* = 130 pupae), 1.0 ± 1.0% after the second release (*n* = 104 pupae), and 4.9 ± 1.6% after the third release (*n* = 185 pupae) (Figure 5). In contrast, the TP of *P. vindemiae* was 32.9 ± 5.2% after the first release (*n* = 82 pupae), 60.2 ± 3.9% after the second release (*n* = 161 pupae), and 35.9 ± 3.7% after the third release (*n* = 170 pupae).

TP differed significantly between the two parasitoid species in all three releases. *P. vindemiae* consistently achieved higher TP than *M. raptor* after the first (χ^2^ = 33.18, df = 1, *p* < 0.001), second (χ^2^ = 95.30, df = 1, *p* < 0.001), and third releases (χ^2^ = 53.84, df = 1, *p* < 0.001).

#### 3.1.3. Emergence Success (ES), Fly Emergence Rate (FER) and Pupal Mortality Rate (PMR)

The values of ES, FER and PMR are summarized in Table 1. When data from the three releases were pooled, Pearson’s χ^2^ analysis revealed significant differences between *M. raptor* and *P. vindemiae* for ES and FER (Table 1), whereas PMR did not differ significantly between the two species overall.

At the release level, no significant differences in ES and FER were detected after the first release, whereas both indices differed significantly between the two parasitoid species after the second and third releases (Table 1). In contrast, PMR differed significantly between the two species in all three releases (Table 1). However, the direction of this difference changed over time: *M. raptor* showed a higher PMR than *P. vindemiae* after the first release, whereas *P. vindemiae* showed a higher PMR than *M. raptor* after the second and third releases.

#### 3.1.4. Longevity (LO)

The mean (±SE) longevity of *M. raptor* was 5 ± 1 days (n = 17). The LO of *P. vindemiae* was about one day shorter than that of *M. raptor*, with a mean value of 4 ± 1 days (n = 47). However, the Mann–Whitney U test did not reveal any statistically significant difference between the longevity of the two parasitoids (U = 42, *p* = 0.619).

#### 3.1.5. Sex Ratio (SR)

The SR (±BSE) of *M. raptor* and *P. vindemiae* were 88.2 ± 7.8% (15 females and 2 males) and 95.7 ± 2.9% (45 females and 2 males), respectively. Pearson’s chi-square (χ^2^) test showed no statistically significant difference between the SR of the two parasitoids (χ^2^ = 1.202, df = 1, *p* = 0.273).

## 4. Discussion

The analysis of parasitization parameters and biological data indicated that both *M. raptor* and *P. vindemiae* were able to kill *P. casei* pupae under conditions typical of ham production facilities. However, although *P. vindemiae* showed a significantly higher ER than *M. raptor*, emergence rates were low in both species. One possible explanation is that, in the absence of a food source, female parasitoids may have increased host feeding activity to acquire nutrients for maintenance and egg maturation at the expense of oviposition [34]. Nevertheless, similarly low ER values have been reported for both species when reared on *P. casei* pupae under laboratory conditions, even when adult parasitoids were provided with honey [11]. Therefore, the low ER observed in the present study is unlikely to be explained solely by the lack of supplementary food. Physiological incompatibility between parasitoid and host, leading to host mortality without successful parasitoid development, may also have contributed to the reduced emergence [25]. This pattern was particularly evident in *P. vindemiae*, which showed high TP despite low ER. These findings are consistent with previous studies reporting that although *P. vindemiae* can oviposit in host puparia at different developmental stages, parasitization of developmentally unsuitable or immature puparia frequently results in progeny mortality despite successful oviposition [25,29].

In this study, the TP index provided information on parasitization performance, offering a clear measure of the potential impact of parasitoid oviposition on the target host. ER provided an evaluation of the establishment capacity of a parasitoid in the host population, in other words, the inoculative potential. Considering these parameters separately may lead to an incomplete assessment of parasitoid performance. Indeed, a parasitoid may cause substantial host mortality while producing relatively few offspring, as observed for *P. vindemiae*. Therefore, integrating TP and ER provides a more realistic evaluation of both the immediate effect on host mortality and the long-term establishment potential of the biological control agent.

In this investigation, LO in the absence of honey was low in both parasitoid species, with mean values of less than a week. This suggests that providing food sources could be important for prolonging their lifespan. However, these data underestimate the longevity, as they do not consider host feeding. Under commercial conditions, increasing adult longevity could extend the period during which females are able to search for and parasitize hosts, potentially improving biological control efficiency. Therefore, providing suitable food sources within the production environment may deserve further investigation. LO was, however, measured under a laboratory photoperiod, whereas the curing room remained dark for most of the time, so the influence of light conditions on adult survival in this environment remains to be established.

Our results demonstrated a strongly female-biased SR for both parasitoid species, consistent with previous studies [24,35]. In haplodiploid parasitoids, female offspring originate from fertilized eggs, and a female-biased sex ratio is therefore consistent with successful mating and sperm transfer [36]. Since mating occurred directly within the ham production facility, these findings indicate that both parasitoid species were able to locate mates and reproduce successfully under operational conditions. From a biological control perspective, SR is an important parameter because only females parasitize hosts and contribute to the control of *P. casei*.

Pupal mortality (PMR) did not differ significantly between the two species overall, despite significant fluctuations in its direction at the release level. Interestingly, the direction of this difference reversed between the first and subsequent releases, suggesting that the progressive establishment of the parasitoid population may have influenced host mortality unrelated to parasitism over the course of the trial. Overall, under the experimental conditions of the present study, *P. vindemiae* showed a higher TP and ER, together with a lower FER, than *M. raptor*, and therefore appears to be a more promising candidate for the biological control of *P. casei*. This finding is consistent with preliminary laboratory bioassays [11]. Since each species was tested in a single cage, the extent to which this difference applies beyond the present system remains to be established.

During the trial, an insecticide treatment was carried out by the ham production facility in the curing room where the customized cages were located, without prior communication to the research team. This unplanned intervention introduced a source of experimental variability that could not be anticipated or controlled. At the same time, because such interventions can occur under real production conditions, this event underscores the practical relevance of our findings and highlights an operational challenge that should be considered when planning future applications of biological control in commercial ham production facilities.

Parasitization by *P. vindemiae* varied among releases, with TP rising from 33% after the first release to 60% after the second and falling to 36% after the third, while ER dropped to 2%. The decline coincides with the insecticide treatment described above. Adult parasitoids are more exposed to residues than host pupae protected within the puparium, and the immature stages developing inside the puparium are themselves susceptible, so that both oviposition and progeny survival would be reduced. The concurrent increase in FER from 9% to 38% indicates that the flies were comparatively unaffected. Since pupae were observed for at least one month, well beyond the 20 to 25 days required for development, the low ER reflects genuine mortality of the immature parasitoid rather than incomplete development. These observations point to a limited capacity of the parasitoid to maintain a stable presence between successive releases, and support the choice of an inundative strategy.

This research supports the need for a multistep approach combining multiple tactics to reduce *P. casei* infestation levels within ham production facilities, particularly in the absence of an acceptable damage threshold for this species in dry-cured ham production [37]. The biological control program using pupal parasitoids could contribute to the long-term suppression of *P. casei* populations by acting on their population dynamics. This approach is particularly well suited to controlled environments such as ham production facilities, where the general absence of non-target organisms minimizes the risk of unintended effects associated with the use of natural enemies for biological control.

Concerning the potential contamination of food products by the released natural enemies, our research showed that pupal parasitoids did not contaminate the hams under the conditions of this study, supporting further investigation under commercial conditions. Inspections after the research found no parasitoids in the experimental ham cages (data provided by the technical staff of the ham production facility). Within the limits of the present study, biological control with pupal parasitoids may deserve investigation as a complement to sanitation practices, with the aim of reducing the growth of *P. casei* populations over successive generations without the need for chemical treatments or prolonged production interruptions.

The present study has structural limitations that should be borne in mind when interpreting the results. The experimental unit was the cage, and one cage was assigned to each parasitoid species, so that the number of true replicates is one per treatment and the comparison between species is confounded with the cage in which each species was tested. The binomial standard errors reported quantify the sampling uncertainty of each estimated proportion given the number of pupae examined, and do not measure empirical variation among independent experimental units. The comparisons reported therefore concern the pupal populations of the cages examined, and the extent to which the differences observed apply to other facilities or populations remains to be established.

Future research should focus on optimizing biological control strategies under the operational conditions of commercial ham production facilities. In particular, the timing and frequency of releases should be adapted to minimize the impact of routine production activities on parasitoid survival and establishment. Although the release interval adopted in this study was based on the developmental time of the parasitoids under laboratory conditions, future studies should investigate whether more frequent releases could improve parasitoid establishment and maintain a more stable parasitoid population over time, thereby enhancing biological control under commercial ham production conditions. Furthermore, evaluating the compatibility of biological control agents with commonly adopted sanitation practices would contribute to improving the practical implementation of these strategies. Taken together, the low ER and LO values observed in this study, combined with the disturbance introduced by the unplanned insecticide treatment, suggest that inundative releases are likely to be more suitable than inoculative releases under commercial conditions.

## 5. Conclusions

This is the first study in which pupal parasitoids have been released against *P. casei* within a dry-cured ham production facility under experimental confinement in customized cages placed inside a curing room. The previously documented capacity for parasitization was maintained in these conditions, and the hams remained free of parasitoids, which is essential for any application in this sector. A replicated design, with several independent cages per parasitoid species and ideally more than one production facility, is the necessary next step. In various sectors of the food industry, the application of biological control has been progressively explored and adopted as the accumulation of scientific and practical evidence has expanded. Preliminary studies, such as the one presented here, are crucial for providing initial proof of using biological control in animal-derived products.

## Figures and Tables

**Table 1:** Values are expressed as percentages. Release and overall values were calculated from pooled counts of pupae and their corresponding outcomes; error values represent the binomial standard error (BSE). Statistical comparisons between parasitoid species were performed using Pearson’s χ^2^ test. FER, fly emergence rate; PMR, pupal mortality rate; ER, parasitoid emergence rate; TP, total parasitism; ES, emergence success. ns, not significant (*p* ≥ 0.05); * *p* < 0.05; *** *p* < 0.001.

Parasitoid	Release	Sample	Pupae	Release Level Values (% ± BSE)					Overall Values (% ± BSE)				
				FER	PMR	ER	TP	ES	FER	PMR	ER	TP	ES
*M. raptor*	1	1	35	55 ± 4%	42 ± 4%	4 ± 2%	4 ± 2%	58 ± 4%	71 ± 2%	25 ± 2%	4 ± 1%	4 ± 1%	75 ± 2%
*M. raptor*	1	2	78										
*M. raptor*	1	3	17										
*M. raptor*	2	1	10	80 ± 4%	19 ± 4%	1 ± 1%	1 ± 1%	81 ± 4%					
*M. raptor*	2	2	49										
*M. raptor*	2	3	45										
*M. raptor*	3	1	38	78 ± 3%	17 ± 3%	5 ± 2%	5 ± 2%	83 ± 3%					
*M. raptor*	3	2	71										
*M. raptor*	3	3	76										
*P. vindemiae*	1	1	20	52 ± 6%	15 ± 4%	9 ± 3%	33 ± 5%	61 ± 5%	29 ± 2%	26 ± 2%	11 ± 2%	45 ± 2%	41 ± 2%
*P. vindemiae*	1	2	26										
*P. vindemiae*	1	3	36										
*P. vindemiae*	2	1	54	9 ± 2%	31± 4%	22 ± 3%	60 ± 4%	31 ± 4%					
*P. vindemiae*	2	2	86										
*P. vindemiae*	2	3	21										
*P. vindemiae*	3	1	63	38 ± 4%	26 ± 3%	2 ± 1%	36 ± 4%	40 ± 4%					
*P. vindemiae*	3	2	60										
*P. vindemiae*	3	3	47										
**Between-species comparisons (Pearson’s χ^2^ test)**				**Release level**					**Overall**				
**Comparison**				**FER**	**PMR**	**ER**	**TP**	**ES**	**FER**	**PMR**	**ER**	**TP**	**ES**
**Release 1**				0.1 ns	17.0 ***	2.1 ns	33.2 ***	0.1 ns	73.1 ***	0.08 ns	18.4 ***	193.5 ***	100.2 ***
**Release 2**				137.7 ***	4.5 *	24.1 ***	95.3 ***	62.5 ***					
**Release 3**				60.7 ***	5.0 *	1.6 ns	53.8 ***	70.7 ***					

## Data Availability

The data presented in this study are available from the corresponding author.

## Abbreviations

The following abbreviations are used in this manuscript:

TP	Total parasitism
ER	Emergence rate
SR	Sex ratio
LO	Longevity
ES	Emergence success
FER	Fly emergence rate
PMR	Pupal mortality rate
BSE	Binomial standard error
